# *Mycobacterium tuberculosis* Cell Wall Antigens Induce the Formation of Immune Complexes and the Development of Vasculitis in an Experimental Murine Model

**DOI:** 10.3390/ijms24021242

**Published:** 2023-01-08

**Authors:** Flaubert Alexis Pérez-Noriega, Citlaltepetl Salinas-Lara, Carlos Sánchez-Garibay, José Jiram Torres-Ruíz, José Luis Maravillas-Montero, Mauricio Castañón-Arreola, María Elena Hernández-Campos, Cesar Rodríguez-Balderas, Beatriz Victoria Basurto-López, Carlos Peñafiel-Salgado, Ana Paola Espinosa-García, José Alberto Choreño-Parra, Martha Lilia Tena-Suck, Luis O. Soto-Rojas, Elsa Y. León-Marroquín, José Pablo Romero-López, Manuel Castillejos-López

**Affiliations:** 1 Departamento de Neuropatología, Instituto Nacional de Neurología y Neurocirugía “Manuel Velasco Suarez”, Mexico City 14269, Mexico; 2 Red MEDICI, Carrera Médico Cirujano, Facultad de Estudios Superiores Iztacala, Universidad Nacional Autónoma de México, Tlalnepantla 54090, Mexico; 3 Tuberculosis Research Commonwealth, Mexico City 14269, Mexico; 4 Laboratorio de Patogénesis Molecular, Laboratorio 4, Edificio A4, Carrera Médico Cirujano, Facultad de Estudios Superiores Iztacala, Universidad Nacional Autónoma de México, Tlalnepantla 54090, Mexico; 5 Departamento de Inmunología y Reumatología, Instituto Nacional de Ciencias Médicas y Nutrición Salvador Zubirán, Mexico City 14080, Mexico; 6 Red de Apoyo a la Investigación, Coordinación de Investigación Científica, Universidad Nacional Autónoma de México, e Instituto Nacional de Ciencias Médicas y Nutrición Salvador Zubirán, Mexico City 04510, Mexico; 7 Genomic Sciences Program, Autonomous University of Mexico City, Mexico City 03100, Mexico; 8 Escuela Superior de Medicina, Sección de Estudios de Postgrado, Instituto Politécnico Nacional, México City 11340, Mexico; 9 Departamento de Bioterio, Instituto Nacional de Neurología y Neurocirugía “Manuel Velasco Suarez”, Mexico City 14269, Mexico; 10 Laboratory of Immunobiology and Genetics, Instituto Nacional de Enfermedades Respiratorias Ismael Cosío Villegas, Mexico City 14080, Mexico; 11 Departamento de Física Médica, Hospital De Oncología, Centro Médico Nacional Siglo XXI, Instituto Méxicano del Seguro Social, Mexico City 06720, Mexico; 12 Departamento de Epidemiología Hospitalaria e Infectología, Instituto Nacional de Enfermedades Respiratorias Ismael Cosio Villegas, Mexico City 14080, Mexico

**Keywords:** *M. tuberculosis*, antigens, cell wall, immunecomplexes, vasculitis, type III hypersensitivity, murine model

## Abstract

Tuberculosis (TB) of the central nervous system (CNS) presents high mortality due to brain damage and inflammation events. The formation and deposition of immune complexes (ICs) in the brain microvasculature during *Mycobacterium tuberculosis* (Mtb) infection are crucial for its pathobiology. The relevance of ICs to Mtb antigens in the pathogenesis of CNS-TB has been poorly explored. Here, we aimed to establish a murine experimental model of ICs-mediated brain vasculitis induced by cell wall antigens of Mtb. We administered a cell wall extract of the prototype pathogenic Mtb strain H37Rv to male BALB/c mice by subcutaneous and intravenous routes. Serum concentration and deposition of ICs onto blood vessels were determined by polyethylene glycol precipitation, ELISA, and immunofluorescence. Histopathological changes in the brain, lung, spleen, liver, and kidney were evaluated by hematoxylin and eosin staining. Our results evidenced that vasculitis developed in the studied tissues. High serum levels of ICs and vascular deposition were evident in the brain, lung, and kidneys early after the last cell wall antigen administration. Cell wall Mtb antigens induce strong type III hypersensitivity reactions and the development of systemic vasculitis with brain vascular changes and meningitis, supporting a role for ICs in the pathogenesis of TB.

## 1. Introduction

Mtb, the causative pathogen of pulmonary TB, affects the lungs and extrapulmonary organs by lymphohematogenous dissemination [1,2,3]. CNS Mtb infection is the most severe and devastating form of TB and has the worst survival due to vascular damage, ischemia, cerebral infarcts, and hydrocephalus [1,4,5,6]. Cerebrovascular events are the most common neurological manifestations of CNS infection as they occur in 15% to 57% of cases of tuberculous meningitis (TBM) [7,8,9]. These complications can originate from vasculitis and endothelial necrosis of the small and medium vessels secondary to direct Mtb infection, with or without an associated prothrombotic state [7,10] or by type III hypersensitivity reactions [11,12,13].

Cases of leukocytoclastic vasculitis and tuberculous glomerulonephritis associated with hypersensitivity reactions to Mtb antigens have been reported. Interestingly, some studies indicate that these reactions coincide with the initiation of anti-TB treatment [14,15,16,17]. Type III hypersensitivity has been seen even among patients with the detectable genetic material of Mtb in vitreous samples who develop idiopathic obliterative retinal vasculitis and Eales disease. Noticeably, these patients have no cultivable bacteria, which supports the notion that the formation of ICs against Mtb antigens and subsequent vasculitis can occur even in the absence of viable bacilli, and not only by direct infection of the endothelium [18]. Hence, type III hypersensitivity reactions could emerge when mycobacterial antigens are released after bacterial lysis.

The deposition of ICs in the microvasculature mediates the activation of the complement system leading to vasculitis of different organs [19]. Furthermore, ICs can be readouts of ongoing anti-TB immune responses. Accordingly, there is evidence that, in pulmonary TB, high serum levels of ICs are observed mainly during active disease [20,21,22,23,24]. Despite their possible relevance in the pathobiology of TB and CNS complications, the pathogenic role of ICs to Mtb antigens has been scarcely investigated. The objective of this study was to address whether antigens of Mtb could induce ICs and the development of vasculitis. For this purpose, we established a novel murine model of type III hypersensitivity induced by the subcutaneous sensitization and subsequent intravenous re-challenge with mycobacterial cell wall antigens. Our results demonstrate that cell wall Mtb antigens induce strong type III hypersensitivity reactions and the development of systemic vasculitis with brain vascular changes and meningitis, supporting a role for ICs in the pathogenesis of TB.

## 2. Results

### 2.1. Administration of Cell Wall Mtb Antigens Induces Cerebral Vasculitis in Mice

**Figure 1:**
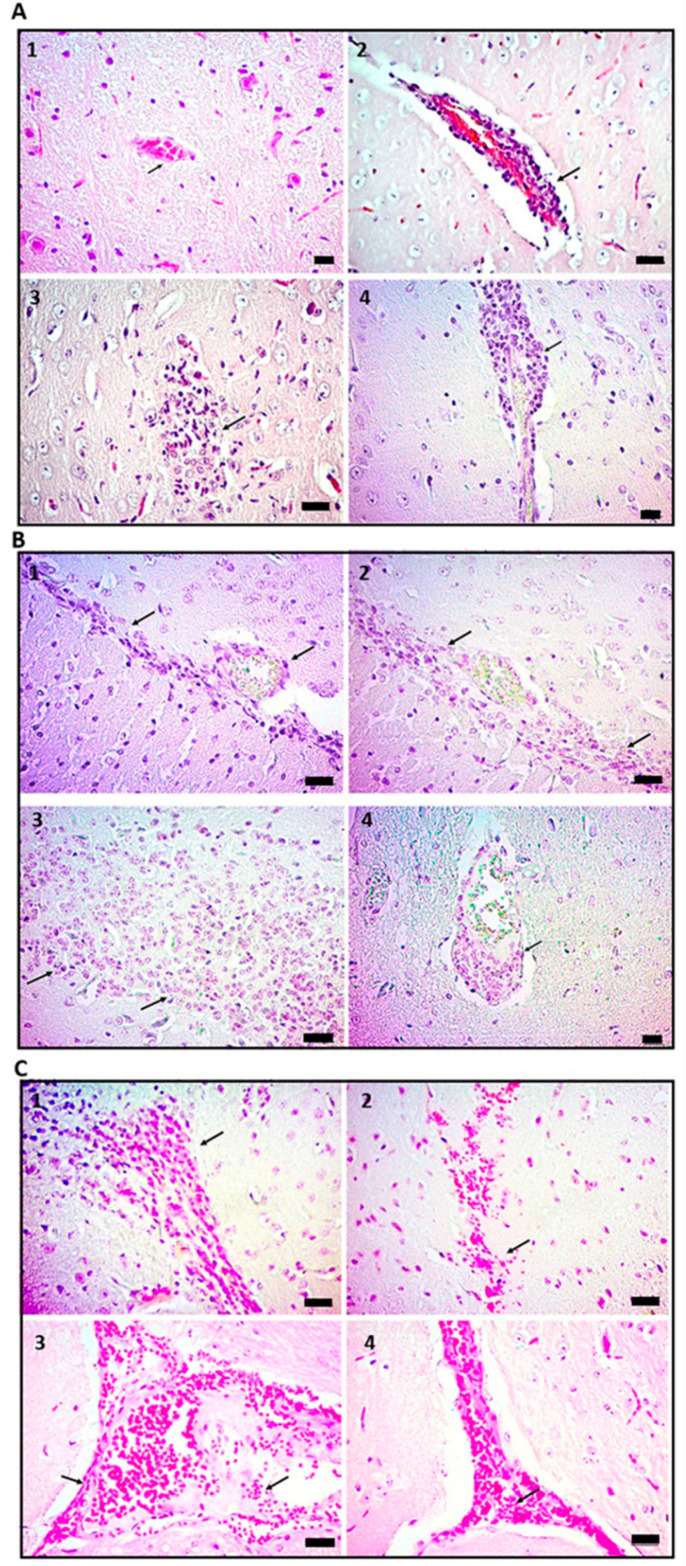
Histopathological features of the brain. (**A1**) control, the arrow point to normal blood vessel; (**A2**,**A3**) vasculitis with perivascular lymphocytic inflammatory infiltrate and cerebral edema on day 1 after the re-challenge with Mtb antigens; (**A4**) vasculitis and cerebral edema on day 14 with mononuclear inflammatory infiltrate, arrows in A2-A4 point to lymphocytic inflammatory infiltrate; (**B1**–**B3**) strong induction of vasculitis, cerebral edema, and meningitis with lymphocytic inflammatory infiltrate on day 14; (**B4**) perivascular lymphocyte infiltration and cerebral edema on day 21, arrows in B1-B4 point to lymphocytic inflammatory infiltrate; (**C1**,**C2**) vasculitis, vascular ectasia, mild cerebral edema, and persistent meningitis on day 28, arrows mark the lymphocytic inflammatory infiltrate and small blood vessel with ectasia; (**C3**,**C4**) focal histopathological changes with vascular ectasia, inflammatory infiltrate, and mild cerebral edema on day 60, arrows point to congested blood vessels. Hematoxylin and eosin stain 40×. Scale bar 20 µm.

To determine the importance of ICs in the pathophysiology of CNS-TB, we analyzed the histopathological changes of different organs harvested from mice that were sensitized and re-challenged with a cell wall preparation of Mtb H37Rv. The histopathological features of the brain tissue showed vasculitis with perivascular lymphocytic inflammatory infiltrate and cerebral edema on day one after the re-challenge with Mtb antigens (Figure 1A2,A3). The most outstanding vascular and cerebral effects occurred mainly on days 14 and 21. As such, on day 14, there was strong induction of vasculitis, cerebral edema, and meningitis with mononuclear inflammatory infiltrate (Figure 1A4,B1–B3). Meanwhile, on day 21, perivascular lymphocyte infiltration and cerebral edema were still observed (Figure 1B4). 

On day 28, the blood vessels showed inflammation with abundant mononuclear cells, vascular ectasia, mild cerebral edema, and persistent meningitis (Figure 1C1,C2). Finally, on day 60, the brain presented focal histopathological changes with vascular ectasia, inflammatory infiltrate, and mild cerebral edema (Figure 1C3,C4). These findings support the idea that vasculitis is a harmful critical mechanism associated with CNS manifestations of TB. 

### 2.2. Vasculitis Induced by Mtb Antigens Is Systemic and Generalizes to Visceral Organs

**Figure 2:**
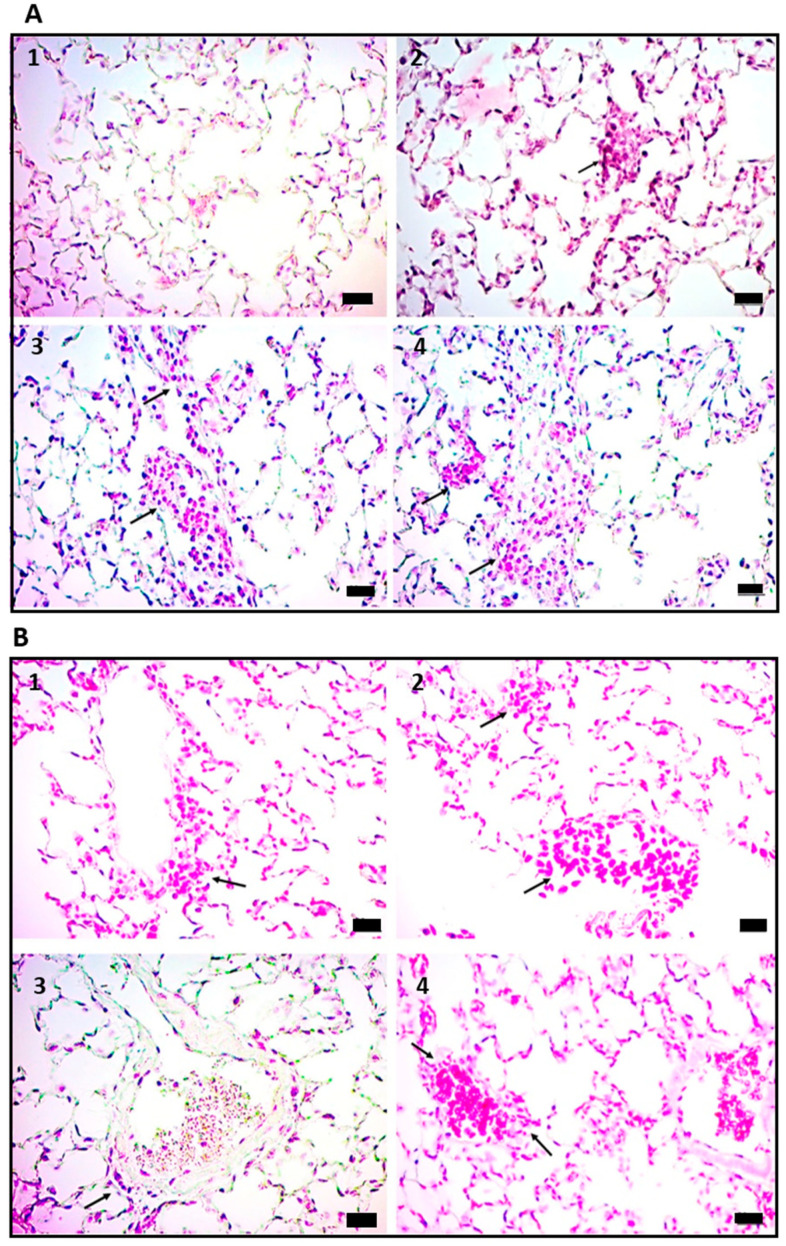
Histopathological features of the lung. (**A1**) Control with normal alveoli; (**A2**) alveolar inflammatory infiltrates with a predominance of macrophages on day 1, arrow point to small granuloma; (**A3**,**A4**) chronic perivascular inflammatory infiltrates in bronchioles and pleura on day 14, arrows mark small blood vessels with ectasia and lymphocytic inflammatory infiltrate; (**B1**,**B2**) mononuclear chronic inflammatory infiltrate-type vasculitis was marked with an arrows in peribronchiolar blood vessels on day 21; (**B3**) vascular ectasia and vasculitis were observed on day 28, pointed with an arrow blood vessel wall thickened; (**B4**) blood vessels with ectasia and adhesion of mononuclear inflammatory cells in the vascular wall on day 60, was signed with an arrow. Hematoxylin and eosin stain 40×. Scale bar 20 µm.

Strikingly, the histopathological changes found in the brain of mice receiving cell wall Mtb antigens were generalized to other organs, including the lungs. Consequently, early alveolar inflammatory infiltrates with a predominance of macrophages were observed in the lung of mice treated with Mtb antigens one day after the second administration (Figure 2A2). On day 14, mouse lungs showed chronic perivascular inflammatory infiltrates in bronchioles and pleura (Figure 2A3,A4). 

On day 21, mononuclear chronic inflammatory infiltrate-type vasculitis was found in peribronchiolar blood vessels (Figure 2B1,B2). On day 28, vascular ectasia and vasculitis were observed (Figure 2B3). On day 60, blood vessels with ectasia and adhesion of mononuclear inflammatory cells in the vascular wall were observed (Figure 2B4).

**Figure 3:**
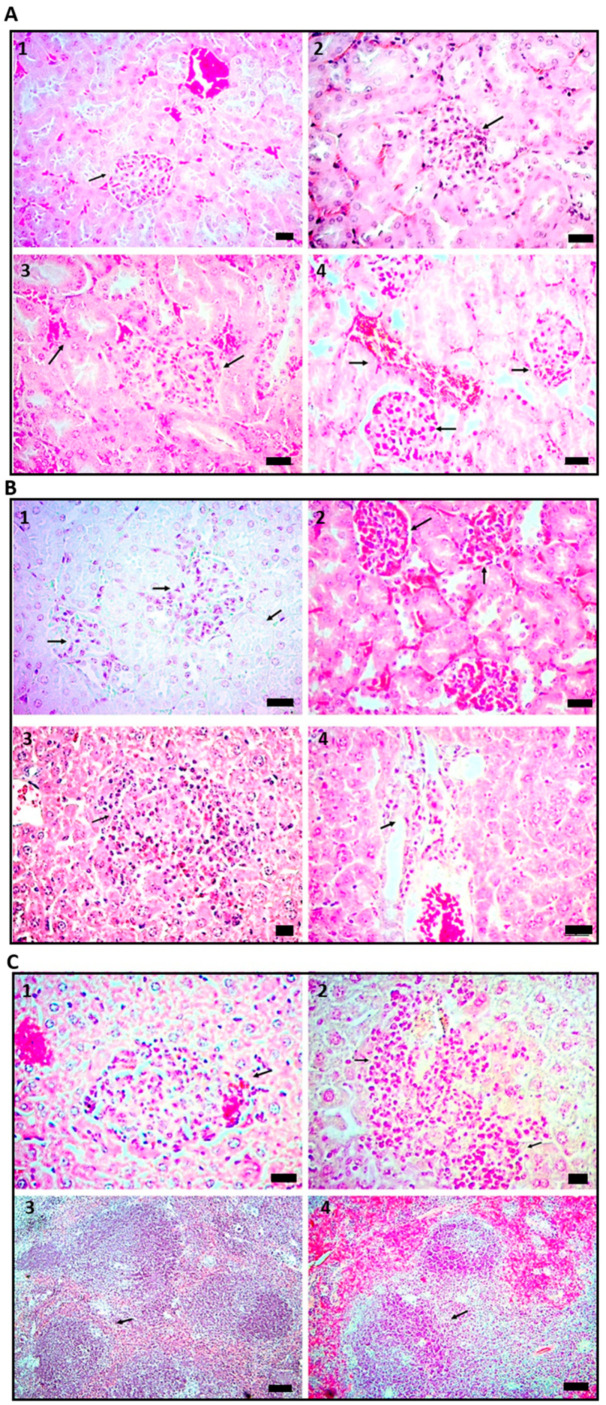
Histopathological features of the kidney, liver, and spleen. (**A1**) control kidney, arrow point to normal glomerulus; (**A2**) slight loss of glomerular architecture, vascular congestion, perivascular and peritubular inflammatory infiltrate is marked with an arrow, on day 1; (**A3**) vascular congestion, there was ectasia, focal glomerular hyperplasia with infiltration of polymorphonuclear cells, and tubular hypertrophy on day 14; (**A4**) hyperplastic glomeruli, neutrophil infiltration, hypertrophic tubules, loss of glomerular architecture, and vascular congestion on day 21, arrows in A3-A4 point to congested blood vessels. (**B1**,**B2**) histopathological features of the kidney on days 28 and 60 were observed as a loss of glomerular architecture, mild inflammatory infiltrates are indicated in B1, vascular congestion is indicated in B2, tubule hypertrophy, and glomerular hyperplasia; (**B3**–**C2**) histopathological features of the liver. B3: arrow point to chronic inflammatory process with mononuclear cell infiltrates on day 1. B4: vascular ectasia is indicated and chronic peri-lobular and perivascular inflammatory infiltrate on day 14; (**C1**,**C2**) evident vasculitis with significant accumulations of lymphocytes and macrophages distributed around the blood vessels with a granuloma-like leukocyte arrangement are indicated in both pictures; Hematoxylin and eosin stain 40× Scale bar 20 µm. (**C3**,**C4**) histopathological features of the spleen; most relevant finding was hyperplasia of the secondary follicles, active germinal center reactions are indicated with arrows, on all evaluated time points we observed the hyperplasic event. Hematoxylin and eosin stain 10×. Scale bar 80 µm.

Similar changes were observed in the kidneys, liver, and spleen. On day 1, the cell wall preparation induced mild perivascular and peritubular inflammatory infiltrate in the kidneys, slight loss of glomerular architecture, and vascular congestion (Figure 3A2). On day 14, in addition to vascular congestion, there was ectasia, focal glomerular hyperplasia with infiltration of polymorphonuclear cells, and tubular hypertrophy (Figure 3A3). On days 21 and 28, hyperplastic glomeruli, neutrophil infiltration, hypertrophic tubules, loss of glomerular architecture, and vascular congestion were found, all suggestive of glomerulonephritis (Figure 3A4,B1). On day 60, there was a loss of glomerular architecture, mild inflammatory infiltrates, vascular congestion, tubule hypertrophy, and glomerular hyperplasia (Figure 3B2). Regarding the changes in the liver, on day one, there was a chronic inflammatory process with mononuclear cell infiltrates (Figure 3B3). On day 14, vascular ectasia and chronic peri-lobular and perivascular inflammatory infiltrate were found (Figure 3B4). On days 21 and 28, evident vasculitis with significant accumulations of lymphocytes and macrophages distributed around the blood vessels with a granuloma-like leukocyte arrangement was observed (Figure 3C1,C2). As in the other viscera, inflamed blood vessels and vascular ectasia were characteristic of the livers of treated mice on day 60 after the last Mtb antigen administration. Finally, in the spleen, the most relevant finding was the hyperplasia of the secondary follicles with evidence of active germinal center reactions on all evaluated time points (Figure 3C3,C4). The rest of the spleen histology was normal, and the control animals showed no histopathological abnormalities in any organ.

### 2.3. Vasculitis Induced by Cell Wall Mtb Antigens Is Mediated by Deposition of ICs

**Figure 4:**
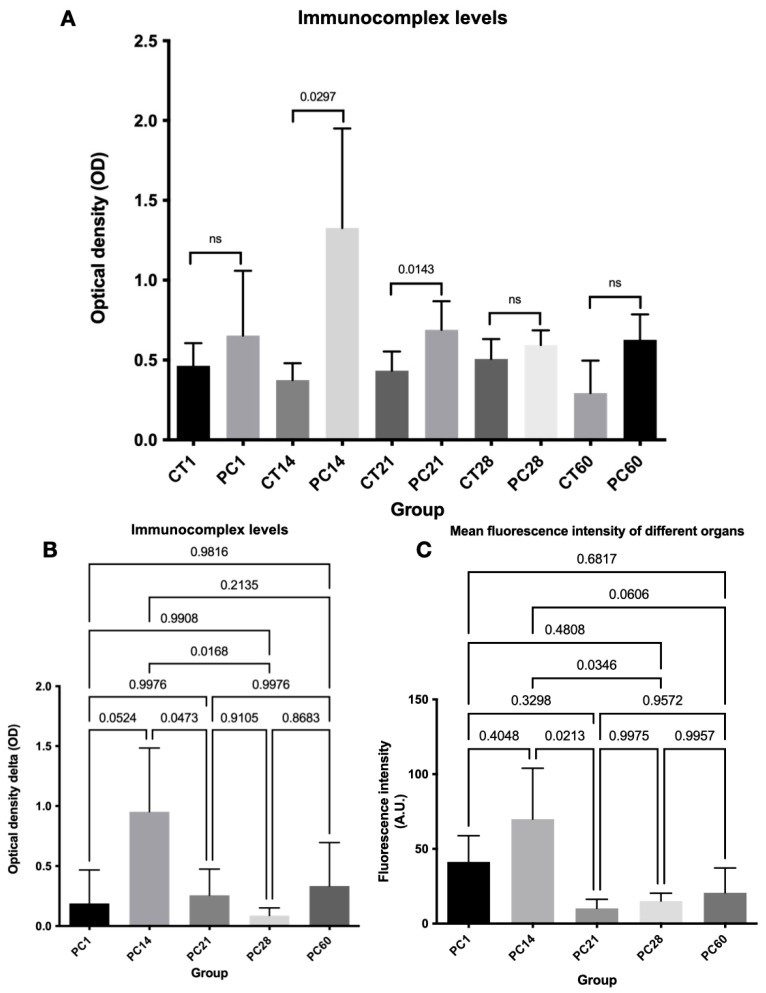
Immunocomplex serum levels were measured by precipitation and ELISA. (**A**) Higher levels of ICs were found in the mice that received the cell wall preparation of Mtb H37Rv, but not in the controls. (**B**) The levels of ICs measured in the control group were subtracted from the experimental group to better track this phenomenon, revealing that the highest relative level of ICs occurred on day 14. (**C**) Mean fluorescence intensity of brain (MFI), lungs, and kidneys. Each tissue microphotograph showed a correlation between the quantitative MFI values and the semiquantitative image interpretations. A high level of MFI was observed on day 14. Abbreviation: CT 1, 14, 21. 28, 60: control of day 1, 14, 21, 28, 60, respectively. ns; no significant difference. PC 1, 14, 21, 28, 60: group that received the cell wall preparation of Mtb H37Rv of day 1, 14, 21, 28, 60, respectively.

**Figure 5:**
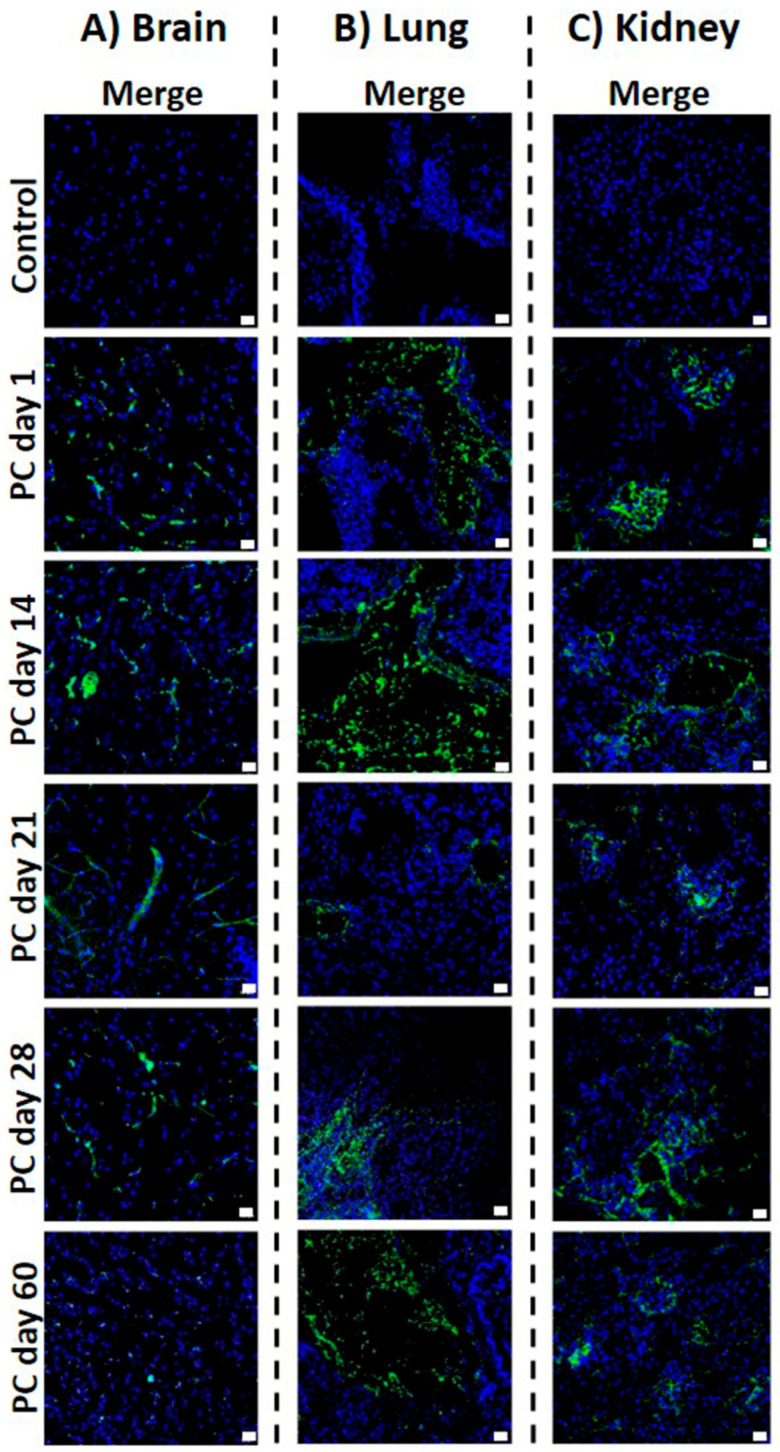
Immunofluorescence characterization of brain, lung, and kidney tissues. (**A**) The deposition of ICs (green channel) in the brain was evident since day one in the mice treated with the cell wall extract, with the MFI peaking on day 14. Thereafter, the MFI progressively decreased, although the deposition of ICs allowed visualization of the vascular trajectories even 21 days. On day 60, the MFI was minimal, and the control group did not show any fluorescence; (**B**) immunofluorescence characterization of lung tissue. The deposition of ICs (green channel) was observed since day one, again being day 14, the time point with the greatest MFI. The fluorescence intensity decreased gradually after day 14, though it was still visible on day 60. The control group did not show any fluorescence. (**C**) ICs (green channel) were deposited in the kidney glomeruli from day 1, peaked on day 14, and slightly decreased on days 28 and 60. Cell nuclei are evident in the blue channel. Scale bar: 10 µm.

Together, the previous findings support the idea that vasculitis is an essential component of the immunopathology of TB and its neurological sequela. To determine whether type III hypersensitivity reactions to Mtb antigens mediated the multiorgan vascular damage observed in our model, we measured the formation of ICs by precipitation and ELISA, as described above. Noticeably, high levels of ICs were found in the mice that received the cell wall preparation of Mtb H37Rv, but not in the controls. This elevation was evident from day one, peaked on day 14 (*p* < 0.03), decreased on day 21(*p* < 0.02), and showed a modest relapse on day 60 (Figure 4A). The levels of ICs measured in the control group were subtracted from the experimental group to better track this phenomenon, revealing that the highest relative level of ICs occurred on day 14 (Figure 4B). Then, using immunofluorescence assays, we evaluated the vascular deposition of ICs. Interestingly, we found that the deposition of ICs in the brain was evident since day one in the mice treated with the cell wall extract, with the mean fluorescence intensity (MFI) peaking on day 14. Thereafter, the MFI progressively decreased, although the deposition of ICs allowed visualization of the vascular trajectories even 21 days after the last mycobacterial cell wall administration. On day 60, the MFI was minimal, and the control group did not show any fluorescence (Figure 5A).

In the lungs, the deposition of ICs was observed since day one, again being day 14, the time point with the greatest MFI. The fluorescence intensity decreased gradually after day 14, though it was still visible on day 60 (Figure 5B). ICs were deposited in the kidney glomeruli from day 1, peaked on day 14, and slightly decreased on days 28 and 60 (Figure 5C). Each tissue microphotograph showed a correlation between the quantitative MFI values and the semiquantitative image interpretations (Figure 4C). Collectively, our results provide a novel animal model of experimental CNS-TB with vasculitis mediated by the formation and deposition of ICs after administration of a cell wall antigen extract of Mtb H37Rv. 

## 3. Discussion

CNS infection is the most devastating form of TB, with vascular lesions characterized by lymphocytic vasculitis, angiodestruction, and cerebral hemorrhage. The origin of this form of extrapulmonary TB is still a matter of intense debate, with most hypotheses suggesting that Mtb enters the CNS before causing local tissue damage. However, live mycobacteria have not been found in biological specimens of cerebral TB, so immunological mechanisms could be involved in the pathophysiology of this disease. Here, we reproduce what occurs in the active phase of tuberculous meningoencephalitis in a murine model, but using only cell wall Mtb antigens. Our findings show that lymphocytic vasculitis, meningeal inflammation, cerebral edema, and angiodestruction are similar to the damage observed among patients with tuberculous meningoencephalitis.

Of note, in mouse tissues of the brain, liver, spleen, kidney, and lung exposed to the mycobacterial cell wall extracts, we observed histopathological changes corresponding to chronic inflammation, where perivascular monocytic infiltrates, macrophage aggregates, and changes derived from tissue repair were evident. These findings resemble those reported in the murine model of experimental progressive pulmonary TB induced by infection with viable Mtb H37Rv [25]. Interestingly, the changes in our model occurred in conditions with no active infection. Therefore, we propose that the cerebrovascular damage of CNS-TB may be mediated by immune responses to Mtb antigens secondarily affecting blood vessels and with subsequent involvement of the contiguous nerve/meningeal tissue. It is noteworthy that this type of perivascular inflammation is also found in pathologies of autoimmune origin, where no microorganism is involved. Two illustrative examples are the neuroinflammation that occurs in systemic lupus erythematosus (SLE) owing to the disruption of the blood–brain barrier (BBB) secondary to the endothelial lesion caused by IC deposition and inflammation, a pathogenic phenomenon behind SLE [26]. Similarly, primary angiitis of the CNS is characterized by aseptic acute necrotizing lymphocytic vasculitis and granulomatous inflammation associated with anti-neutrophil cytoplasmic antibodies [27,28]. Thus, it is not unreasonable to assume that Mtb antigens induce aseptic encephalitis and vasculitis, probably due to the vascular deposition of ICs and inflammation, an event that can also increase BBB permeability.

Some studies in Lewis rats treated intravenously with antibodies to the Mtb mannan have shown BBB permeability without histopathological changes in the brain. These findings suggest that mannan induces strong antibody responses that facilitate the BBB opening and the induction of experimental allergic encephalomyelitis. However, these studies have not clarified the precise mechanism of this phenomenon [29]. In this context, our findings expand on this topic, adding evidence that implicates type III hypersensitivity reactions to cell wall Mtb antigens as the underlying mechanism causing BBB permeability, aseptic cerebral encephalitis, and vasculitis.

An outstanding observation of our study was that the histopathological changes induced by Mtb antigens in the brain generalized also to the lungs, kidneys, and liver. These changes consisted of perivascular chronic inflammatory infiltrates associated with the deposition of ICs, as observed by IFs. Hence, cell wall components of Mtb can provoke systemic type III hypersensitivity such as in other conditions such as SLE, vasculitis, serum disease, skin lesions, and even bacterial infectious diseases [30]. Intriguingly, similar to our results, ICs-induced changes in glomerular architecture, glomerular hyperplasia, and vascular congestion are observed in proliferative focal segmental glomerulonephritis in the kidney. Glomerular damage affects renal function by altering the glomerular-tubular balance leading to hypertrophy of the renal tubules. These findings have been reported in TB cases that develop vasculitis and glomerulonephritis during Mtb infection [14,15,31]. In addition, type III hypersensitivity reactions have been described in patients with pulmonary TB who develop cutaneous vasculitis after starting treatment with rifampicin and pyrazinamide [32,33]. These data further support our hypothesis that the formation of ICs is not only induced by active infection with live mycobacteria, but after bacterial lysis and en masse releasing of Mtb antigens as well [20,22,34,35,36].

The dynamics of the formation and deposition of ICs in our study showed a bimodal behavior with increasing ICs levels detected from day 1 to day 14 after re-challenge (acute phase), and a slight rebound on day 60 (chronic phase). This kinetic pattern coincides with the dynamics of plasma ICs and circulation of B cell-secreting antibodies specific to lipoarabinomannans and the 38-kDa Mtb antigen during the first two weeks of anti-TB treatment in patients with active pulmonary TB [37]. This pattern could be a consequence of the release of mycobacterial antigens at the sites of infection and in peripheral blood, with a later decrease according to the progress of the treatment and the bactericidal activity of anti-TB drugs. Thus, we suggest that the acute phase of this response occurs due to: (1) antigen availability and (2) the dynamics of the humoral immune response. The subsequent decrease in IC levels that we observed may be due to their solubilization and purification, although as shown by IF, there is still vascular deposition. The late relapse in ICs levels on day 60 could be explained by the dynamics of memory B cell and plasma cell differentiation, which can secrete specific antibodies and survive without antigenic stimulation [38,39]. Alterations leading to constant activation of the complement system may play a role as well, and other authors have shown that, in active pulmonary TB patients, there are also abnormalities in the solubilization of ICs leading to persistent aseptic complement activation [40]. Moreover, ICs may be formed in situ in the endothelium by specific antibodies towards endothelial molecules, which react by molecular mimicry with cell wall Mtb antigens [41,42]. The specific mechanism leading to chronic ICs deposition and vasculitis in TB must be investigated in future studies. 

Finally, limitations of our study include that the precise isotype of the ICs detected in mice treated were not determined with cell wall Mtb antigens. In this regard, other reports indicate that the antibodies that form ICs during active TB are mostly IgGs [19,34,43]. Furthermore, we did not characterize the biochemical nature of the cell wall Mtb antigens inducing ICs in our model. In patients with pulmonary TB, proteomic analysis shows that the majority of Mtb antigens forming ICs are proteins of the extracellular region of Mtb involved in immune evasion and enzyme modulators [34]. Therefore, the subsequent should better characterize the specific Mtb antigens that elicit ICs formation.

In summary, our study supports the idea that cell wall Mtb antigens released by bacterial lysis induce ICs and systemic vasculitis by triggering type III hypersensitivity reactions. These harmful mechanisms might be involved in the development of neurological complications of pulmonary TB. Finally, we provided a novel animal model that could be useful to study new therapeutic strategies to counteract the consequences of ICs in TB and other disorders mediated by type III hypersensitivity responses.

## 4. Materials and Methods

### 4.1. Experimental Animals

Male BALB/c mice were bred at the animal facility of the Instituto Nacional de Neurología y Neurocirugía “Manuel Velasco Suárez” (INNyN) and used at 8–10 weeks of age (weight 20–25 g). The mice were housed in polypropylene boxes, maintained at an ambient temperature of 21 °C, with a photoperiod of 12 h of light and 12 h of darkness, with free air, food, and water on demand. Mice were weighed upon receipt and allocated into subgroups of five mice per box, according to the groups mentioned below. The mice were acclimatized for 48 h before starting the experiments. The animal procedures were evaluated and authorized by the Institutional Committee for the Care and Use of Laboratory Animals of the INNyN (CICUAL-INNyN) under project number 28/19.

### 4.2. Mtb Cell Wall Preparations

Mycobacterial cell wall extracts were obtained by a modification of the protocol proposed by Hirschfield et al. [44]. Briefly, frozen bacteria of the H37Rv Mtb strain were cultured to the late logarithmic phase in a culture medium with salt, glycerol, and alanine. The bacilli were suspended in PBS with eight mM EDTA (8993-50, J.T.Baker, PA, USA, DNase I RNase free, (FEREN0521, Thermo Fisher™, Waltham, MA, USA)., RNase A (1901, Qiagen, Hilden, Germany), and PMSF (1087091001 Sigma™ MO, United States) as proteinase inhibitors (rupture buffer). For cell fragmentation, 0.1-mm glass beads were added to the suspension of bacilli in rupture buffer, and lysis was completed with a high-energy cell disruptor in five cycles of 60 s with 42 W of power. Intact cells that did not fragment were removed by low-speed centrifugation at 3000× *g*. The supernatant was passed through a 0.22-µm syringe filter and centrifuged at 22,000× *g* for 1 h. The precipitate was dissolved in 1 mM PBS-EDTA and centrifuged at 27,000× *g* for 1 h. The sediment corresponding to the cell wall was resuspended and dialyzed in 10 mM ammonium bicarbonate (1066337 Sigma™ MO, United States) to a final cell wall concentration of 1 µg/µL.

### 4.3. Exposure of Mice with the Mtb Cell Wall

Experimental mice were administered with 25 µL of the cell wall preparation plus 25 µL of incomplete Freund’s adjuvant on day zero (sensitization) by subcutaneous injection and on day 15 intravenously in the caudal vein (re-challenge). A separate group of mice were administered with 0.9% saline solution (PiSA^®^, Hidalgo, México) by the same injection routes in parallel with experimental animals and served as controls. The mice were euthanized by anesthetic overdose with sodium pentobarbital (PiSA^®^, Hidalgo, México) on days 1, 14, 21, 28, or 60 after the second immunization (n = 5 per group). Blood was collected by intracardiac puncture, and the spleen, brain, liver, lung, and kidney were dissected and processed for histopathological analysis.

### 4.4. Quantification of Immune Complexes

The immune complexes were purified by the precipitation technique using the polyethylene glycol polymer of 8000 Da (PEG-8000, Sigma™ MO, United States) at 7% and 3.5%, following the method of Ranganathan et al. [45]. Then, 7% PEG-8000 was added to the serum at a 1:1 volume ratio. The mixture was incubated at 4 °C overnight, and then samples were centrifuged at 13,000 rpm at 4 °C for 10 min. The supernatants were discarded, and the precipitates were washed with 3.5% PEG-8000. Finally, the precipitate was diluted in PBS. The immune complexes were quantified by the sandwich ELISA technique using a goat anti-mouse IgG (H + L) antibody and a secondary Ab HRP 62-6520 (Thermo Fisher™, Waltham, MA, USA). The optical density (OD) values of the control group were subtracted from the values of the experimental group.

### 4.5. Histopathological Analysis

The harvested murine organs were processed with the conventional histological technique for hematoxylin and eosin (H&E) staining. The slides of the histological sections were observed with a bright-field binocular microscope, and the control and experimental groups were compared to tissue morphology.

### 4.6. Evidence of IC Deposition

Deposition of ICs was detected by direct immunofluorescence (IF) using a primary antibody that is part of the immune complex associated with cell wall Mtb antigens and a secondary Alexa Fluor 488 donkey anti-mouse IgG (H + L) antibody (Thermo Fisher™, Waltham, MA, USA) diluted in 5% bovine serum albumin (Sigma™ St. Louis, Missouri, United States) at. The chromatin was stained with Hoechst 33342 (Thermo Fisher™ Waltham, MA, USA), and the coverslips were mounted with ProLong™ Gold Antifade Mountant (Thermo Fisher™ Waltham, MA, USA). The photomicrographs were taken with a Nikon Ti-E confocal microscope (Minato, Tokyo, Japan), and the mean fluorescence intensity (MFI) was quantified using the free software Fiji 2.9.0 (ImageJ ™, MD, USA) by taking the average of six fields at 40×.

### 4.7. Statistical Analysis

Descriptive and inferential statistics were performed in Prism 8.4.2 (GraphPad, La Jolla, CA, USA). Descriptive statistics and the Shapiro–Wilk test were used to verify the normality of the dataset, and the parametric hypothesis tests analysis of variance and Student’s *t*-test were run to analyze the data obtained. Statistical significance was established at *p* < 0.05.

## Data Availability

The data that support the findings of this study are available from the corresponding author upon reasonable request.
